# Recovery of Renal Function among ESRD Patients in the US Medicare Program

**DOI:** 10.1371/journal.pone.0083447

**Published:** 2013-12-17

**Authors:** Sumit Mohan, Edwin Huff, Jay Wish, Michael Lilly, Shu-Cheng Chen, William M. McClellan, for the Fistula First Breakthrough Initiative Data Committee

**Affiliations:** 1 Division of Nephrology, Department of Medicine, Columbia University College of Physicians & Surgeons, New York, New York, United States of America; 2 Division of Quality Improvement, Centers for Medicare & Medicaid Services, Boston Regional Office, Boston, Massachusetts, United States of America; 3 Division of Nephrology, Department of Medicine, Case Western Reserve University, Cleveland, Ohio, United States of America; 4 Division of Vascular Surgery, Department of Surgery, University of Maryland Baltimore School of Medicine, Baltimore, Maryland, United States of America; 5 Chronic Disease Research Group, Minneapolis Medical Research Foundation, Minneapolis, Minnesota, United States of America; 6 Division of Nephrology, Department of Medicine, Emory School of Medicine, Atlanta, Georgia, United States of America; University of Florida, United States of America

## Abstract

**Background:**

Patients started on long term hemodialysis have typically had low rates of reported renal recovery with recent estimates ranging from 0.9–2.4% while higher rates of recovery have been reported in cohorts with higher percentages of patients with acute renal failure requiring dialysis.

**Study Design:**

Our analysis followed approximately 194,000 patients who were initiated on hemodialysis during a 2-year period (2008 & 2009) with CMS-2728 forms submitted to CMS by dialysis facilities, cross-referenced with patient record updates through the end of 2010, and tracked through December 2010 in the CMS SIMS registry.

**Results:**

We report a sustained renal recovery (i.e no return to ESRD during the available follow up period) rate among Medicare ESRD patients of > 5% - much higher than previously reported. Recovery occurred primarily in the first 2 months post incident dialysis, and was more likely in cases with renal failure secondary to etiologies associated with acute kidney injury. Patients experiencing sustained recovery were markedly less likely than true long-term ESRD patients to have permanent vascular accesses in place at incident hemodialysis, while non-White patients, and patients with any prior nephrology care appeared to have significantly lower rates of renal recovery. We also found widespread geographic variation in the rates of renal recovery across the United States.

**Conclusions:**

Renal recovery rates in the US Medicare ESRD program are higher than previously reported and appear to have significant geographic variation. Patients with diagnoses associated with acute kidney injury who are initiated on long-term hemodialysis have significantly higher rates of renal recovery than the general ESRD population and lower rates of permanent access placement.

## Introduction

Recovery of renal function in patients requiring prolonged hemodialysis is thought to be a relatively uncommon occurrence. Large observational cohorts of patients started on long term hemodialysis from different parts of the world reported renal recovery rates of as low as 1%-2.4%[1,2] . Since 2007, the United States Renal Data System(USRDS) has excluded from its definition of prevalent end stage renal disease (ESRD) patients who experienced recovery of renal function occurring within the first 180 days of first ESRD service and persisting for at least 90 days – and has applied this definition retroactively[1]. However, during this period of exclusion, the rate of renal recovery has been estimated, for the 1995 - 2006 period, to be approximately 0.9% of the ESRD population and more recent data has suggested a rapid increase in the rate of recovery in the US ESRD program [3,4]. Smaller cohort studies have suggested slightly higher rates of recovery among incident peritoneal dialysis patients[5]. A higher rate is reported in patient cohorts that include a large percentage of patients who are dialysis-dependent following acute kidney injury (AKI)[2]. For example, in a recent large observational cohort of patients admitted to the ICU who required renal replacement therapy, 78% of patients experienced renal recovery within one year[6]. 

Our primary hypothesis was that sustained recovery of renal function is more likely to occur with diagnoses that are associated with AKI. We expected that patients with temporaryrecovery of renal function— i.e., those who recovered and later returned to hemodialysis — were likely to have underlying chronic kidney disease with either progression that was temporarily reversible or with superimposed acute kidney injury that improved, and thus were excluded from our primary analysis. These patients were analyzed as a separate subgroup of the cohort. We also studied the geographic variation in the overall rates of renal recovery and the rates of renal recovery from AKI across the continental United States. 

## Methods

### Data Source and patient cohort

All patients with end stage renal disease who enroll for Medicare ESRD benefits are required to have an ESRD Medical Evidence Report (“2728 form”) submitted within 45 days of initiation of therapy by their dialysis or transplant providers to the Centers for Medicare & Medicaid Services (CMS)[7]. Physicians completing the form are required to certify that the patient has “reached the stage of renal impairment that appears irreversible and permanent and requires a regular course of dialysis or kidney transplant to maintain life.” Instructions accompanying the form explicitly state, “This form SHOULD NOT be completed for those patients who are in acute renal failure. Acute renal failure is a condition in which kidney function can be expected to recover after a short period of dialysis, i.e., several weeks or months.”[7] We obtained Medicare ESRD registration data from CMS-2728 forms submitted to CMS by dialysis facilities for calendar years 2008 and 2009. Data from these forms were cross-referenced with patient record updates through the end of 2010 to identify patient status changes due to renal recovery. Patients who died following initiation of dialysis were included in our cohort and follow up was censored at the time of death. 

We identified a total of 194,007 incident hemodialysis patients who were thought to have met the criteria for the ESRD benefit at the time of registration and began hemodialysis in 2008 or 2009, and were tracked through December 31, 2010. CMS contracts with 18 geographically based ESRD Networks that cover the United States and territories, to support and improve the quality of care to beneficiaries in the Medicare ESRD program, interfacing between ESRD care providers and CMS. The CMS Standard Information Management System (SIMS) database receives verified information from the 18 ESRD Networks on a monthly basis. 

### Definitions

#### Sustained Recovery

Incident ESRD patients registered in 2008 and 2009 in their SIMS registry record, who had recovery of renal function, as indicated by patient event code “9,” and did not return for either dialysis or transplantation through the end of our follow up period of at least one year, and up to three years, were defined as patients with sustained recovery of renal function. 

#### Temporary Recovery

Patients with temporary recovery of renal function, i.e., those patients with recovery of renal function who subsequently returned to hemodialysis within our follow up period, were excluded from our primary analysis of patients with sustained recovery of renal function. It should be noted that 2009 incident patients had at least one year where they were at risk of returning to dialysis, and while this exposure period is a shorter period than for 2008 incident patients, both groups had longer periods of return exposure risk than the 90 days used by the USRDS^1^.

### Statistical Analysis

 For univariate analyses, categorical variables were compared using the chi square test, while continuous variables were compared using the *t* test. A multivariable logistic regression model using patient characteristics that were likely to be associated with sustained renal recovery including demographics (age, gender, race), prior nephrology care, type of dialysis access available primary diagnoses and reported comorbidities. Statistical analyses and map generation were performed using Stata 11.2 (Stata Corp., College Station, TX).

## Results

**Figure 1:**
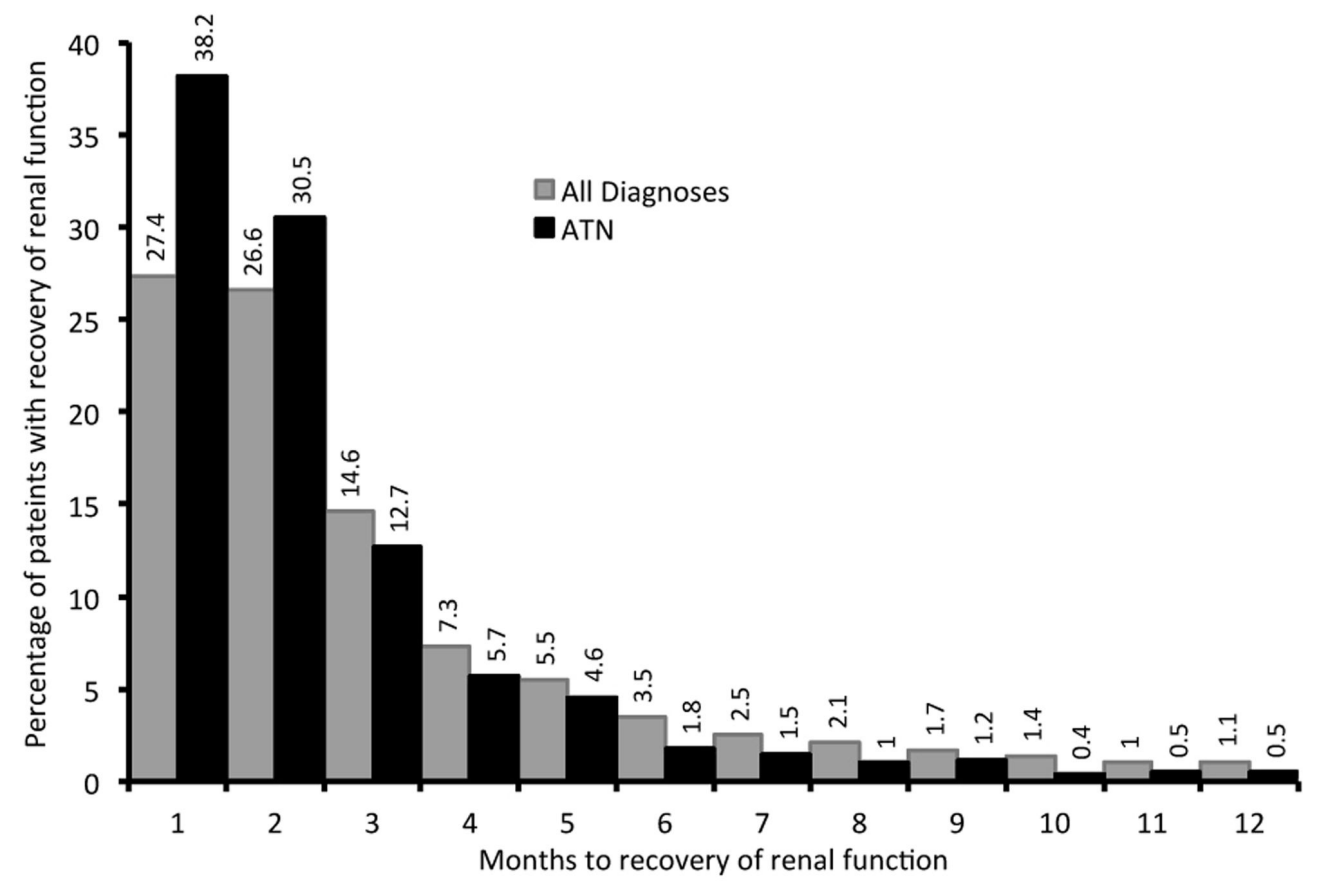
Histogram showing time to renal recovery for the entire cohort and for those patients with ATN – for those patients who do have sustained recovery of renal function.

In the overall cohort of 194,007 patients initiating hemodialysis in 2008 and 2009, we identified 12,970 patients (6.69%) who had evidence of recovery of renal function during the follow up period (Figure 1) . Of these, 1,916 patients (14.8%) with evidence of renal recovery returned to dialysis within the follow-up period (temporary recovery). The rate of sustained renal recovery was significantly higher in 2009 than in 2008 (5.9% vs. 5.6%, *p=0.01*), a difference that persisted even after exclusion of those patients whose ESRD diagnosis was acute tubular necrosis (ATN), the most common diagnosis among those who recovered kidney function. This observation seems consistent with other analyses that have also suggested recent rising rates of renal recovery in the US ESRD program[4]. The rate of sustained recovery showed a similar increase from 2008 to 2009 after exclusion of all diagnoses primarily associated with AKI (2009 vs. 2008: 4.4 vs. 4.2%). 

Patients with evidence of sustained renal recovery were younger on average and more likely to be male than individuals who did not experience any recovery of renal function (Table 1). The racial composition of the groups with and without sustained recovery differed significantly (Table 1), with a higher percentage of patients reported as “white” on the CMS-2728 form in the sustained recovery group than in the group without recovery of renal function. Patients with recovery had similar overall rates of recovery across types of prior insurance coverage, although differences were seen with lower rates of recovery for those covered by Medicare and Medicaid, and higher rates of recovery for those covered by insurance through their employer (Table 1). 

**Table 1:** Comparisons of patients without recovery of renal function with those who have evidence of sustained recovery of renal function as well as that of patients with temporary and sustained recovery of renal function.

**Characteristic**	**ESRD patients without renal recovery**	**ESRD patients with sustained renal recovery**	***p value^1^***	**With temporary Renal Recovery**	***p value^2^***
**N**	**181,037 (94.25%)**	**11,054 (5.68%)**		**1916 (0.99%)**	
Age (years)	63.9 ± 15.6	62.3 ± 15.6	*<0.001*	63.4 ± 14.8	*0.004*
Male (%)	56.5	58.3	*<0.001*	57.7	*0.425*
**Race**					
White (%)	64.6	79.0	*<0.001*	74.7	*<0.001*
Black (%)	29.9	18.0		22.6	
Other (%)	5.6	3.0		2.7	
**Access at Incident dialysis initiation (%)**					
AV fistula (%)	14.8	1.9	*<0.001*	7.41	*<0.001*
AV graft (%)	3.39	0.9		1.25	
Catheter (%)	80.9	96.4		90.08	
Other (%)	0.9	0.8		1.25	
**Insurance coverage**					
Medicaid	11.94	10.27	*<0.001*	11.06	*<0.001*
Veterans Affairs	1.09	0.75		1.2	
Medicare	15.1	14.2		16.34	
Medicare advantage	4.97	4.95		5.01	
Medicare and Medicaid	12.4	9.86		11.95	
Medicare & employer based	7.69	7.35		9.19	
Medicare and other	13.75	14.18		14.93	
Employer based	14.19	18.84		13.62	
Other	6.46	7.22		6.78	
Other combinations	4.64	4.17		4.75	
No insurance coverage	7.76	8.21		5.17	
**Nephrology care prior to ESRD therapy**					
< 6 months (%)	21.1	26.7	*<0.001*	22.42	*<0.001*
6 - 12 months (%)	36.4	40.7		38.05	
> 12 months (%)	42.5	29.7		39.52	
**Comorbid conditions**					
Diabetes (%)	33.57	23.43	<0.001	41.39	<0.001
Hypertension (%)	76.34	65.26	<0.001	76.93	<0.001
Congestive heart failure (%)	29.78	22.08	<0.001	36.95	<0.001
Cerebrovascular accident (%)	8.5	6.59	<0.001	9.19	<0.001
No comorbid conditions reported (%)	0.02	0.1	<0.001	0	0.167

^1^ Comparison between patients with sustained recovery of renal function and no recovery of renal function

^2^ Comarpison of patients with temporary and sustained recovery of renal function

Patients with recovery of renal function had very low rates of permanent accesses (arteriovenous fistula or graft) in place or awaiting maturation at the time of dialysis initiation. This finding also supports our acute etiology hypothesis given that it takes time to place permanent accesses. Similarly, higher usage rates of catheters (over 96%, from Table 1), which can be more quickly placed, would be expected for those in whom renal failure suddenly occurred. 

Several diagnoses were associated with a significantly higher rate of recovery of renal function than the overall rate of sustained renal recovery (5.75%) including acute interstitial nephritis (AIN), acute tubular necrosis (ATN), post partum renal failure, rapidly progressive glomerulonephritis (RPGN), exposure to analgesics and other nephrotoxic agents, cholesterol emboli, hepatorenal syndrome (HRS), and hemolytic uremic syndrome (HUS)(Table 2). ATN alone accounted for 19.9% of all cases of renal recovery (and 22% of all cases of sustained recovery), while other diagnoses that are commonly associated with acute kidney injury - AIN, RPGN, HRS, HUS, post-infectious glomerulonephritis, cholesterol emboli, trauma, liver transplant and toxin exposure – accounted for a further 11.1% of the observed renal recovery cases. These results strongly support our acute hypothesis. 

**Table 2:** Primary renal diagnoses with higher than average percentage of patients with sustained recovery of renal function.

**Primary cause of renal failure**	**ESRD patients with sustained renal recovery (%)**	p value*
Acute interstitial nephritis	42.7	*<0.001*
Acute tubular necrosis	37.0	*<0.001*
Postpartum renal failure	31.4	*<0.001*
Post-infectious glomerulonephritis	30.0	*<0.001*
Hemolytic uremic syndrome	27.0	*<0.001*
Nephropathy secondary to heroin/other drugs	24.2	*<0.001*
Lymphoma of the kidneys	24.2	*<0.001*
Trauma/surgical loss of kidney	23.5	*<0.001*
Benign urinary tract tumors	20.0	*<0.001*
Other nephrotoxic agents	19.1	*<0.001*
Wegner’s granulomatosis	16.7	*<0.001*
Cholesterol emboli	15.9	*<0.001*
Analgesic abuse	15.7	*<0.001*
Other renal disorders	15.5	*<0.001*
Rapidly progressive glomerulonephritis	15.0	*<0.001*
Radiation nephritis	15.0	*<0.001*
Henoch-Schonlein	14.6	*<0.001*
Multiple myeloma	14.0	*<0.001*
Tuberous sclerosis	12.7	*<0.001*
Hepatorenal syndrome	12.8	*<0.001*
Complications of liver transplant	10.4	*<0.001*
Unknown	10.4	*<0.001*
Scleroderma	9.1	*<0.001*
SLE nephritis	8.8	*<0.001*
AIDS nephropathy	8.1	*<0.001*

^*^ *compared to the renal recovery rate in the entire cohort*.

Overall, patients with recovery were only half as likely to have received any prior nephrology care (61.6% vs. 30.6% with rrf). Among those who did receive care from a nephrologist prior to starting dialysis, patients with recovery of renal function were less likely to have had long term care (>12 months), and more likely to have received only recent care (< 6 months) (Table 1). Both of these findings are consistent with our acute etiology hypothesis in that acute onset of renal failure is less likely to afford the necessary time for a specialist referral or consult, so fewer occur, and when they do occur, their duration is shorter.

**Figure 2:**
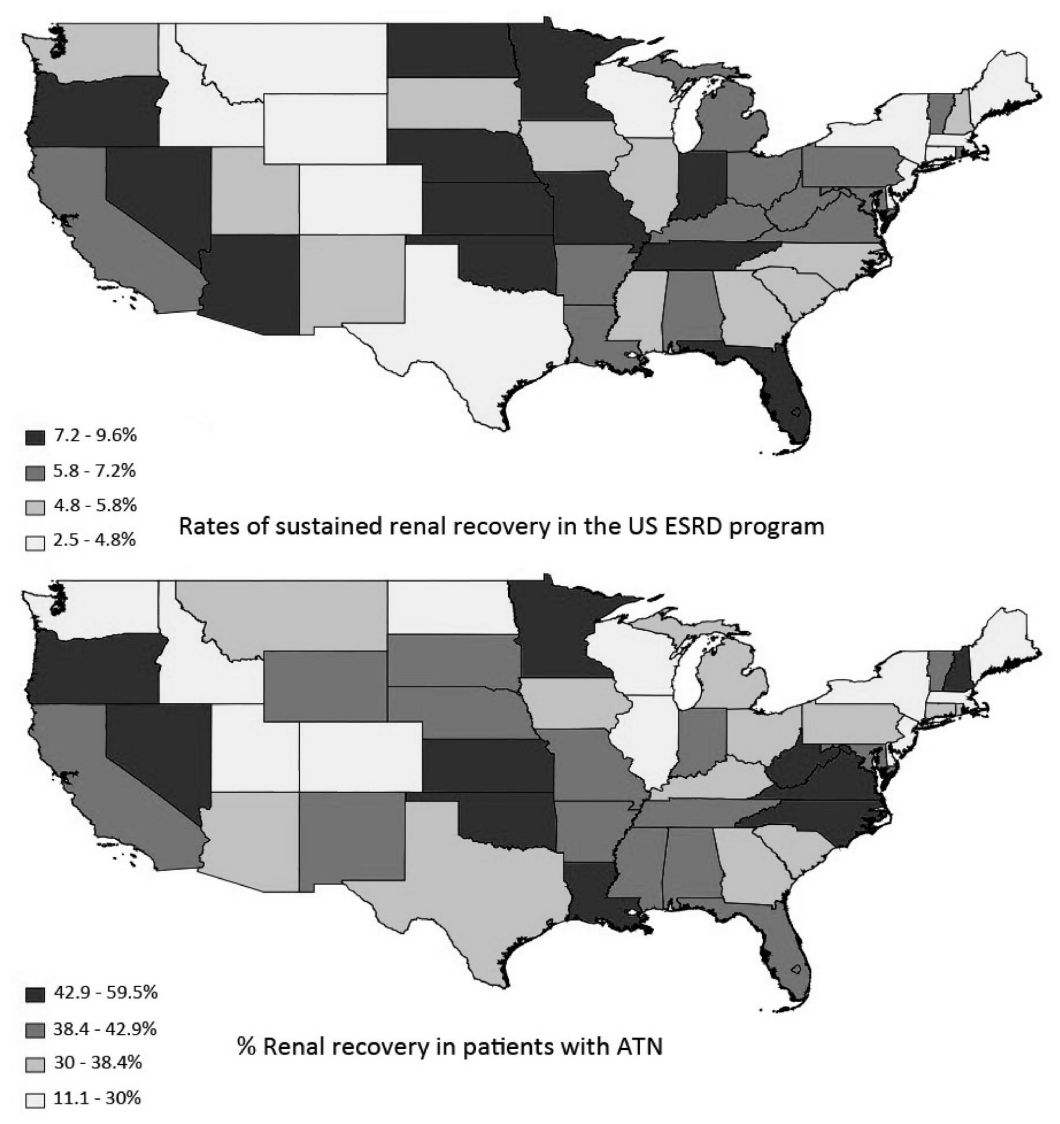
Maps demonstrating the significant geographical variation in the overall rates of renal recovery and the rates of renal recovery for patients initiated on hemodialysis with a diagnosis of “tubular necrosis”.

We also found significant variation in the rates of renal recovery across ESRD Networks and states (Figure 2). The overall rate of renal recovery varied significantly across ESRD Networks, ranging from 3.4% (Network 3) to 7.6% (Network 7). Similarly, there was considerable variation across states, with rates ranging from 2.5% (Delaware) to 9.6% (Kansas). Of note, Guam, the Northern Mariana Islands and American Samoa each had lower rates of renal recovery than Delaware. Further analysis of the relative distribution of various diagnoses demonstrated marked geographic variation at the state or Network level in the rate of renal recovery only for patients with ATN (Figure 2). For example, while more than a third of cases of ATN nationwide experienced recovery of renal function, there was substantial variation among ESRD Networks, ranging from about a quarter of cases experiencing recovery (23.5% in Network 3) to almost half of all cases (48% in Network 13). Sustained recovery of renal function among patients with ATN varied from 0% (Alaska) to 59.5% (Oklahoma).

**Figure 3:**
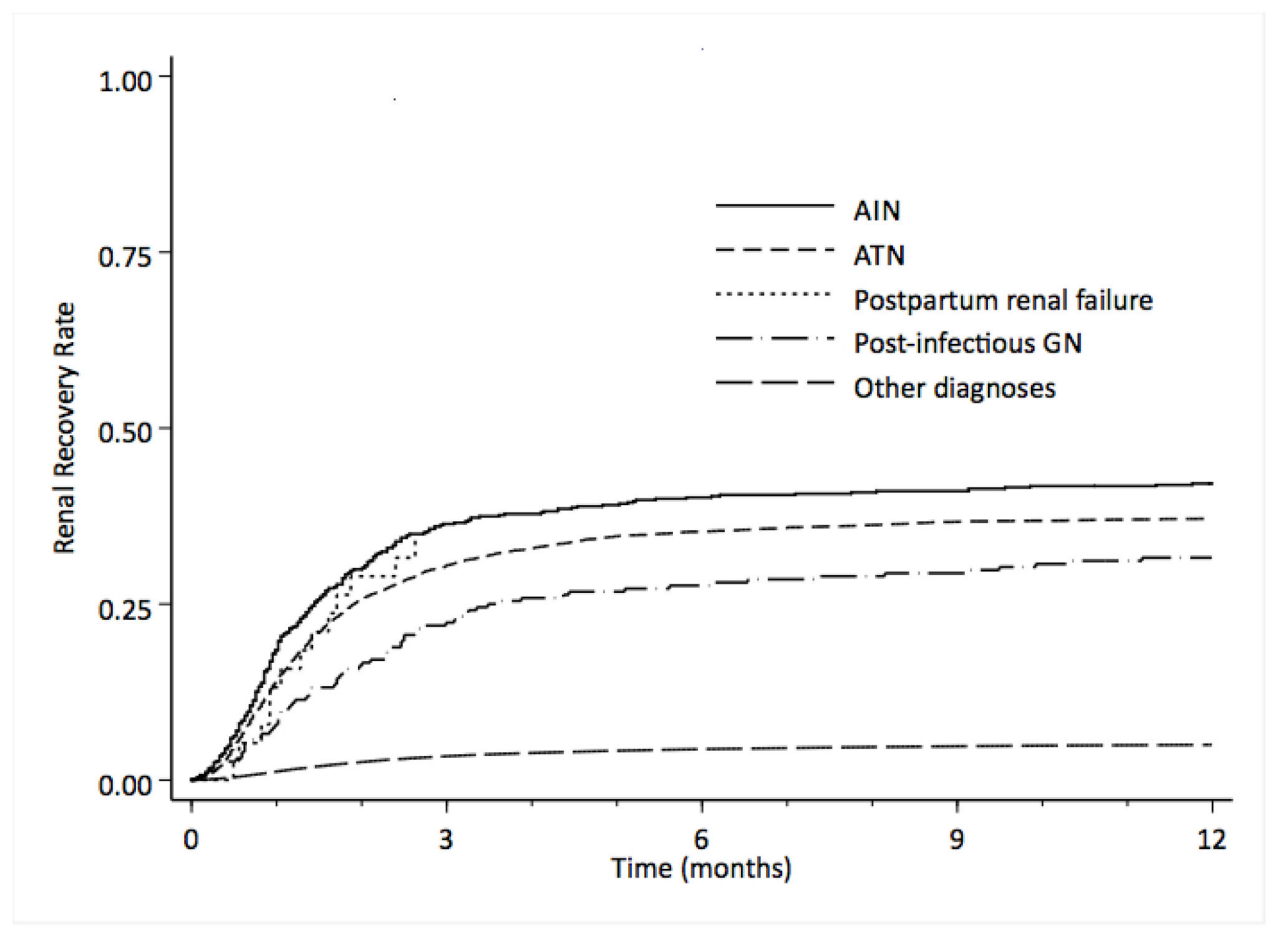
Kaplan Meier curves showing comparison of the rates of sustained recovery of renal function by diagnosis among Medicare ESRD patients.

Renal recovery was most likely to occur in the first month after incident hemodialysis, with more than half the cases of renal recovery occurring within the first two months, and more than two thirds in the first 3 months of registration of ESRD (Figure 1). Rates of recovery for patients with ATN as the primary cause of renal failure were more rapid, with more than two-thirds of cases of renal recovery occurring within the first two months of registration (Figure 1). Kaplan Meier curves for various diagnoses demonstrate that the vast majority of renal recovery occurs within the first few months (Figure 3). These findings of time to recovery, also, strongly support our acute etiology hypothesis.

The multivariable model demonstrated a significantly lower likelihood of renal recovery associated with the placement of a permanent access and among non-white patients (Table 3). Previous care from a nephrologist for any length of time was associated with a lower likelihood of recovery - suggesting that Nephrologist contact was more likely to be associated with patients experiencing a slower, chronic disease process which afforded more time at least for long term access planning. Additionally, a higher likelihood of renal recovery was seen in patients with a history of toxin exposure (“toxic nephropathy”), alcohol/drug abuse and among those patients with no other comorbidities at the time of incident dialysis - findings that are also consistent with the acute etiology hypothesis. 

**Table 3:** Multivariable model for the odds of sustained renal recovery.

	**Odds Ratio**	**Std. Err.**	**z**	**P>|z|**	**95% Confidence Interval**
Age (years)	0.994	0.001	-8.72	0.000	0.993 - 0.995
**Gender**					
Female	Ref				
Male	1.046	0.022	2.12	0.034	1.003 - 1.090
**Access**					
Non-permanent access	Ref				
Permanent access^	0.253	0.015	-22.84	0.000	0.225 - 0.285
**Race**					
White or Other	Ref				
Black	0.551	0.015	-22.02	0.000	0.523 - 0.581
**Primary diagnosis**					
Acute interstitial nephritis	8.459	0.800	22.56	0.000	7.027 - 10.182
Acute tubular necrosis	6.469	0.226	53.47	0.000	6.041 - 6.927
Nephropathy *	3.463	0.320	13.43	0.000	2.889 - 4.152
Cholesterol emboli	2.767	0.300	9.38	0.000	2.237 - 3.424
Multiple myeloma	1.837	0.129	8.64	0.000	1.600 - 2.109
Unknown etiology	1.454	0.064	8.54	0.000	1.334 - 1.585
Hepatorenal syndrome	1.289	0.127	2.56	0.010	1.062 - 1.565
Hypertension	0.711	0.023	-10.63	0.000	0.667 - 0.757
Diabetes	0.544	0.017	-19.10	0.000	0.511 -0.579
**Nephrology Care**					
No prior nephrology care	Ref				
Any prior nephrology care	0.374	0.009	-40.72	0.000	0.356
**Comorbidities**					
Diabetes	0.920	0.025	-3.07	0.002	0.873 - 0.970
Hypertension	0.823	0.019	-8.31	0.000	0.786 - 0.862
COPD	0.933	0.196	-0.33	0.741	0.617 - 1.409
Current smoker	0.876	0.241	-0.48	0.629	0.511 - 1.501
Cancer	1.124	0.208	0.63	0.527	0.782 - 1.614
Toxic nephropathy	7.767	3.975	4.01	0.000	2.849 - 21.180
Alcohol and/or drug abuse	2.315	0.765	2.54	0.011	1.211 - 1.424
Needs assistance**	1.109	0.094	1.22	0.223	0.939 -1.310
Cardiac disease***	0.817	0.020	-8.43	0.000	0.780 -0.856
No comorbidities reported	2.148	0.828	1.99	0.047	1.010 - 4.571

^ Permanent access refers to mature AV fistula or graft only

* Nephropathy caused by agents other than lead and analgesics

** Needs assistance includes institutionalized patients and those needing assistance ambulating and/or transferring

*** Patients with any reported cardiac or vascular disease including congenital heart disease, atherosclerotic heart disease, peripheral vascular disease, cerebrovascular disease, congestive heart failure and/or other cardiac disease

## Discussion

Recovery of renal function among patients requiring hemodialysis is not rare, with multiple reports in the literature[1-4]. Rates of recovery among large cohorts are generally low – in the 1-2% range but there are some suggestions of increasing rates of recovery within the US ESRD program[1-4]. While previous estimates of renal recovery in the Medicare ESRD program were of the same magnitude our analysis suggests the possibility of an increasing rate of renal recovery over time, and also indicates that almost a third of patients who experienced renal recovery have a primary diagnosis for the cause of renal failure that is associated with acute kidney injury[4]. However, an acute insult superimposed on advanced chronic kidney disease could easily result in renal failure requiring dialysis with little hope for sustained recovery. We limited our primary analysis to those patients who experienced recovery of renal failure and did not return to the ESRD program during the follow up period, allowing for a more conservative estimate of the rate of recovery of renal function. Despite this, our analysis suggests a higher rate of renal recovery in the Medicare ESRD program than the previously reported 0.9% rate of recovery of renal function for patients enrolled in the program[8]. 

Specific primary diagnoses that are usually associated with acute kidney injury were seen more commonly among recovered patients: for example, after diabetes and hypertension, the most frequent primary diagnoses were unknown etiology (4.1%) and ATN (3.5%) - which had sustained renal recovery rates of 10.4% and 37% respectively. Other diagnoses associated with acute kidney injury that were included in the top 10 pre-ESRD diagnoses in the cohort included multiple myeloma (14% sustained recovery) and “other renal disorders” (15.5% sustained recovery).

Identification of renal recovery for patients receiving hemodialysis requires careful monitoring on the part of healthcare providers. Thus, perhaps the increase in rates and the geographic variation in renal recovery identified in the Medicare ESRD program may be a reflection of improved identification of recovery of renal function and improved care among hemodialysis patients, rather than changes in ESRD registration patterns. While the dataset precluded us from being able to test this hypothesis, the extremely low rates of permanent access placement in patients who experienced sustained recovery of renal function suggests an expectation of possible, eventual recovery on the part of the treating physician – or may perhaps reflect the absence of nephrology care prior to initiation of chronic hemodialysis. Further, although higher rates of renal recovery could be a direct reflection of changing ESRD patient case-mix created by enrolling increasing percentages of patients with renal failure caused by etiologies that may be reversible, the significant increase in the rate of renal recovery in 2009 compared to 2008 persisted even after exclusion of ATN cases. The rate of renal recovery in 2009 were also marginally higher after exclusion of all the diagnoses primarily associated with AKI (ATN, AIN, RPGN, HRS, HUS, post-partum renal failure, trauma, nephrotoxic agents, post-infectious GN and liver transplant). Therefore, it is possible that the increasing rate of renal recovery are due to factors related to improved patient care such as better attention to fluid weight management and avoidance of intradialytic hypotension, avoidance of nephrotoxins, and better care of the underlying illnesses due to advances in pharmacotherapeutics. Patients who experienced recovery of renal function were also less likely to have permanent access placed suggesting possible recognition for the potential for recovery on the part of the treating physician. As a result, perhaps these patients should be excluded from prevalence estimates for arteriovenous fistulas among ESRD patients in the US. 

Patients with temporary recovery of renal function were older, more likely to be Black and more likely to have Medicare, prior nephrology care, diabetes and hypertension than those who had sustained reocovery of renal function. Temporary recovery of renal function is the result of one of two possible clinical scenarios. Given the differences listed above, the more likely possibility is that patients with acute kidney injury superimposed on advanced CKD, are likely to return to dialysis eventually even if they recover the renal function lost in the acute setting because of the progressive nature of the underlying CKD. The other, less likely, possibility is that patients experience an episode of acute injury requiring dialysis that is followed by recovery and then a subsequent independent/unrelated instance of dialysis requiring loss of renal function. 

While we are able demonstrate significant variations in state and ESRD Network level renal recovery rates, the dataset does not help ascertain if these variations are related to varying physician decision making thresholds for initiation of hemodialysis and registration for ESRD benefits, or to differences in monitoring of patients for evidence of recovery of renal function among registered ESRD patients receiving hemodialysis. The inability to provide outpatient maintenance renal replacement therapy for patients without end stage renal disease appears to be unique to the United States. This may have impacted our analysis and may limit the potential generalizability of our findings to ESRD programs in other countries particularly to programs in countries with universal health care coverage. 

## Conclusions

Rate of recovery of renal function in the Medicare ESRD program appears to be higher than previously reported and appear to be increasing over time. There is marked geographic variation in rates of renal recovery across states and ESRD Networks, particularly for patients reported to have ATN as the primary cause of ESRD. Not surprisingly, patients with diagnoses that are associated with acute kidney injury who are initiated on long-term hemodialysis have significantly higher rates of renal recovery than the general ESRD population. Sustained renal recovery appears to occur predominantly within the first 3 months of initiation of hemodialysis, and continues to be seen over the first 12 months, albeit less frequently. Patients who have recovery of renal function were also less likely, perhaps appropriately, to have permanent access placement at the time of initiation of hemodialysis. As a result, patients with these renal diagnoses at the time of registration are likely to benefit from closer monitoring of their residual renal function, interventions to avoid additional renal injury and periodic reassessment of their need for continued renal replacement therapy. 
